# Supplementary material for: Discovery and application of insertion-deletion (INDEL) polymorphisms for QTL mapping of early life-history traits in Atlantic salmon
Source: BMC Genomics. 2010 Mar 8;11:156. doi: 10.1186/1471-2164-11-156 (PMC2838853; doi:10.1186/1471-2164-11-156)
Supplement: Additional file 2 — Information on developed 76 locus single-run INDEL panel in Atlantic salmon. Information on fluorescence labeling, primer concentrations, PCR pooling and links to alignments, INDEL motifs and GENESCAN (Burge and Karlin 1997) predictions of genes/exons are available in html format. [file 1471-2164-11-156-S2.ZIP › Additionalfile2/snpsummary32.html]

```
Cluster 3 Contig 17

prev  Summary    Contig List  next
```

Size of Consensus sequence = 1161

Number of sequences = 185

Minimum redundancy = 6

Key

A gi|117532319|gb|EG863764.1|EG863764 EST\_ssal\_eve\_48886 ssaleve thyroid Salmo salar cDNA Salmo salar cDNA clone ssal\_eve\_566\_216\_fwd 3', mRNA sequence  
B gi|117867641|gb|EG940337.1|EG940337 EST\_ssal\_evf\_23872 ssalevf mixed\_tissue Salmo salar cDNA Salmo salar cDNA clone ssal\_evf\_531\_208\_fwd 3', mRNA sequence  
C gi|117548982|gb|EG880427.1|EG880427 EST\_ssal\_eve\_23745 ssaleve thyroid Salmo salar cDNA Salmo salar cDNA clone ssal\_eve\_532\_138\_fwd 3', mRNA sequence  
D gi|84208950|gb|DW180300.1|DW180300 SGP303926 Atlantic salmon Gills II cDNA library Salmo salar cDNA clone SS0195F02 5', mRNA sequence  
E gi|84214835|gb|DW183229.1|DW183229 SGP309201 Atlantic salmon White muscle II cDNA library Salmo salar cDNA clone SS0123F10 5', mRNA sequence  
F gi|45314765|gb|CK885134.1|CK885134 SGP165500 Atlantic salmon Intestine cDNA library Salmo salar cDNA clone T4-0412 5', mRNA sequence  
G gi|85020617|gb|DW549273.1|DW549273 EST\_ssal\_rgb2\_13692 rgb2 Salmo salar cDNA clone ssal\_rgb2\_522\_326\_rev 5', mRNA sequence  
H gi|56732983|gb|CX154251.1|CX154251 ssalsla011044\_0 spleen Salmo salar cDNA, mRNA sequence  
I gi|117530467|gb|EG861923.1|EG861923 EST\_ssal\_eve\_55021 ssaleve thyroid Salmo salar cDNA Salmo salar cDNA clone ssal\_eve\_575\_001\_fwd 3', mRNA sequence  
J gi|45315463|gb|CK885832.1|CK885832 SGP166217 Atlantic salmon Intestine cDNA library Salmo salar cDNA clone T5-0255 5', mRNA sequence  
K gi|117852850|gb|EG925546.1|EG925546 EST\_ssal\_evf\_28148 ssalevf mixed\_tissue Salmo salar cDNA Salmo salar cDNA clone ssal\_evf\_537\_123\_fwd 3', mRNA sequence  
L gi|22033921|gb|AJ425753.1|AJ425753 AJ425753 Salmo salar spleen adult Salmo salar cDNA clone s23B05, mRNA sequence  
M gi|117454032|gb|EG786251.1|EG786251 EST\_ssal\_evd\_6945 ssalevd thymus Salmo salar cDNA Salmo salar cDNA clone ssal\_evd\_507\_381\_fwd 3', mRNA sequence  
N gi|29315417|gb|CB504191.1|CB504191 ssalplnb502026\_rev gut Salmo salar cDNA, mRNA sequence  
O gi|29325828|gb|CB514602.1|CB514602 ssalrgb502076\_rev mixed\_tissue Salmo salar cDNA, mRNA sequence  
P gi|85156512|gb|DW589157.1|DW589157 sbra1-008AD12.y1\_#0551.2 DIAS\_SBRA Salmo salar cDNA 5', mRNA sequence  
Q gi|84972405|gb|DW470806.1|DW470806 SGP313438 Atlantic salmon Testis cDNA library Salmo salar cDNA clone MG4-4195 5', mRNA sequence  
R gi|117456705|gb|EG788924.1|EG788924 EST\_ssal\_evd\_9351 ssalevd thymus Salmo salar cDNA Salmo salar cDNA clone ssal\_evd\_511\_100\_fwd 3', mRNA sequence  
S gi|117825367|gb|EG898063.1|EG898063 EST\_ssal\_evf\_37157 ssalevf mixed\_tissue Salmo salar cDNA Salmo salar cDNA clone ssal\_evf\_549\_197\_fwd 3', mRNA sequence  
T gi|84984865|gb|DW535215.1|DW535215 EST\_ssal\_plnb\_3851 plnb Salmo salar cDNA clone ssal\_plnb\_021\_324\_rev 5', mRNA sequence  
U gi|22033225|gb|AJ425057.1|AJ425057 AJ425057 Salmo salar spleen adult Salmo salar cDNA clone s02G11, mRNA sequence  
V gi|29327132|gb|CB515906.1|CB515906 ssalrgb503157\_rev mixed\_tissue Salmo salar cDNA, mRNA sequence  
W gi|117838806|gb|EG911502.1|EG911502 EST\_ssal\_evf\_13510 ssalevf mixed\_tissue Salmo salar cDNA Salmo salar cDNA clone ssal\_evf\_516\_223\_fwd 3', mRNA sequence  
X gi|89841976|gb|DY699165.1|DY699165 EST\_ssal\_rgb2\_54904 ssalrgb2 mixed\_tissue Salmo salar cDNA Salmo salar cDNA clone ssal\_rgb2\_588\_279\_rev 5', mRNA sequence  
Y gi|117858086|gb|EG930782.1|EG930782 EST\_ssal\_evf\_32861 ssalevf mixed\_tissue Salmo salar cDNA Salmo salar cDNA clone ssal\_evf\_543\_264\_fwd 3', mRNA sequence  
Z gi|117458007|gb|EG790226.1|EG790226 EST\_ssal\_evd\_10523 ssalevd thymus Salmo salar cDNA Salmo salar cDNA clone ssal\_evd\_512\_334\_fwd 3', mRNA sequence  
a gi|85055963|gb|DW584141.1|DW584141 EST\_ssal\_rgb2\_48560 rgb2 Salmo salar cDNA clone ssal\_rgb2\_578\_165\_rev 5', mRNA sequence  
b gi|117821057|gb|EG893753.1|EG893753 EST\_ssal\_evf\_51279 ssalevf mixed\_tissue Salmo salar cDNA Salmo salar cDNA clone ssal\_evf\_568\_244\_fwd 3', mRNA sequence  
c gi|117865790|gb|EG938486.1|EG938486 EST\_ssal\_evf\_22206 ssalevf mixed\_tissue Salmo salar cDNA Salmo salar cDNA clone ssal\_evf\_529\_087\_fwd 3', mRNA sequence  
d gi|117869763|gb|EG942459.1|EG942459 EST\_ssal\_evf\_25782 ssalevf mixed\_tissue Salmo salar cDNA Salmo salar cDNA clone ssal\_evf\_534\_053\_fwd 3', mRNA sequence  
e gi|117481373|gb|EG813590.1|EG813590 EST\_ssal\_evd\_33551 ssalevd thymus Salmo salar cDNA Salmo salar cDNA clone ssal\_evd\_544\_260\_fwd 3', mRNA sequence  
f gi|84205710|gb|DW178542.1|DW178542 SGP305294 Atlantic salmon Brain II cDNA library Salmo salar cDNA clone SS0167D11 5', mRNA sequence  
g gi|45308983|gb|CK879352.1|CK879352 SGP143525 Atlantic salmon Gills cDNA library Salmo salar cDNA clone G5-0752 5', mRNA sequence  
h gi|56732869|gb|CX154193.1|CX154193 ssalsla005044\_0 spleen Salmo salar cDNA, mRNA sequence  
i gi|22033365|gb|AJ425197.1|AJ425197 AJ425197 Salmo salar spleen adult Salmo salar cDNA clone s06E07, mRNA sequence  
j gi|85029984|gb|DW558640.1|DW558640 EST\_ssal\_rgb2\_23059 rgb2 Salmo salar cDNA clone ssal\_rgb2\_537\_175\_rev 5', mRNA sequence  
k gi|85018713|gb|DW547369.1|DW547369 EST\_ssal\_rgb2\_11788 rgb2 Salmo salar cDNA clone ssal\_rgb2\_519\_355\_rev 5', mRNA sequence  
l gi|85054039|gb|DW582217.1|DW582217 EST\_ssal\_rgb2\_46636 rgb2 Salmo salar cDNA clone ssal\_rgb2\_575\_151\_rev 5', mRNA sequence  
m gi|117525622|gb|EG857349.1|EG857349 EST\_ssal\_eve\_50905 ssaleve thyroid Salmo salar cDNA Salmo salar cDNA clone ssal\_eve\_569\_125\_fwd 3', mRNA sequence  
n gi|117862487|gb|EG935183.1|EG935183 EST\_ssal\_evf\_19232 ssalevf mixed\_tissue Salmo salar cDNA Salmo salar cDNA clone ssal\_evf\_524\_093\_fwd 3', mRNA sequence  
o gi|117856688|gb|EG929384.1|EG929384 EST\_ssal\_evf\_31604 ssalevf mixed\_tissue Salmo salar cDNA Salmo salar cDNA clone ssal\_evf\_541\_372\_fwd 3', mRNA sequence  
p gi|117472619|gb|EG804838.1|EG804838 EST\_ssal\_evd\_5674 ssalevd thymus Salmo salar cDNA Salmo salar cDNA clone ssal\_evd\_506\_102\_fwd 3', mRNA sequence  
q gi|24464112|gb|BQ036731.1|BQ036731 SS2-0437 Atlantic Salmon spleen SSH Salmo salar cDNA clone SS2-0437 similar to MHCII-like protein, mRNA sequence  
r gi|117446640|gb|EG778859.1|EG778859 EST\_ssal\_evd\_5357 ssalevd thymus Salmo salar cDNA Salmo salar cDNA clone ssal\_evd\_505\_318\_fwd 3', mRNA sequence  
s gi|117858705|gb|EG931401.1|EG931401 EST\_ssal\_evf\_33418 ssalevf mixed\_tissue Salmo salar cDNA Salmo salar cDNA clone ssal\_evf\_544\_168\_fwd 3', mRNA sequence  
t gi|117541958|gb|EG873403.1|EG873403 EST\_ssal\_eve\_34255 ssaleve thyroid Salmo salar cDNA Salmo salar cDNA clone ssal\_eve\_546\_203\_fwd 3', mRNA sequence  
u gi|40542343|gb|BU694030.1|BU694030 EST0067 Atlantic salmon Lambda Zap Express eye cDNA library Salmo salar cDNA clone EER2-e04 5' similar to MHC class II invariant chain-like protein 2, mRNA sequence  
v gi|117854876|gb|EG927572.1|EG927572 EST\_ssal\_evf\_29972 ssalevf mixed\_tissue Salmo salar cDNA Salmo salar cDNA clone ssal\_evf\_539\_298\_fwd 3', mRNA sequence  
w gi|117854278|gb|EG926974.1|EG926974 EST\_ssal\_evf\_29434 ssalevf mixed\_tissue Salmo salar cDNA Salmo salar cDNA clone ssal\_evf\_539\_016\_fwd 3', mRNA sequence  
x gi|117443460|gb|EG775683.1|EG775683 EST\_ssal\_evd\_50899 ssalevd thymus Salmo salar cDNA Salmo salar cDNA clone ssal\_evd\_568\_215\_fwd 3', mRNA sequence  
y gi|117845170|gb|EG917866.1|EG917866 EST\_ssal\_evf\_56976 ssalevf mixed\_tissue Salmo salar cDNA Salmo salar cDNA clone ssal\_evf\_576\_158\_fwd 3', mRNA sequence  
z gi|117490246|gb|EG822463.1|EG822463 EST\_ssal\_evd\_24198 ssalevd thymus Salmo salar cDNA Salmo salar cDNA clone ssal\_evd\_531\_168\_fwd 3', mRNA sequence  
A gi|117858557|gb|EG931253.1|EG931253 EST\_ssal\_evf\_33285 ssalevf mixed\_tissue Salmo salar cDNA Salmo salar cDNA clone ssal\_evf\_544\_100\_fwd 3', mRNA sequence  
B gi|117852917|gb|EG925613.1|EG925613 EST\_ssal\_evf\_28209 ssalevf mixed\_tissue Salmo salar cDNA Salmo salar cDNA clone ssal\_evf\_537\_154\_fwd 3', mRNA sequence  
C gi|117440897|gb|EG773120.1|EG773120 EST\_ssal\_evd\_48592 ssalevd thymus Salmo salar cDNA Salmo salar cDNA clone ssal\_evd\_565\_126\_fwd 3', mRNA sequence  
D gi|29322958|gb|CB511732.1|CB511732 ssalrgb532015\_rev mixed\_tissue Salmo salar cDNA, mRNA sequence  
E gi|117845517|gb|EG918213.1|EG918213 EST\_ssal\_evf\_57289 ssalevf mixed\_tissue Salmo salar cDNA Salmo salar cDNA clone ssal\_evf\_576\_322\_fwd 3', mRNA sequence  
F gi|89850200|gb|DY706323.1|DY706323 EST\_ssal\_rgb2\_62062 ssalrgb2 mixed\_tissue Salmo salar cDNA Salmo salar cDNA clone ssal\_rgb2\_599\_320\_rev 5', mRNA sequence  
G gi|117834966|gb|EG907662.1|EG907662 EST\_ssal\_evf\_10053 ssalevf mixed\_tissue Salmo salar cDNA Salmo salar cDNA clone ssal\_evf\_511\_308\_fwd 3', mRNA sequence  
H gi|89850867|gb|DY706990.1|DY706990 EST\_ssal\_rgb2\_62729 ssalrgb2 mixed\_tissue Salmo salar cDNA Salmo salar cDNA clone ssal\_rgb2\_601\_122\_rev 5', mRNA sequence  
I gi|85049531|gb|DW577709.1|DW577709 EST\_ssal\_rgb2\_42128 rgb2 Salmo salar cDNA clone ssal\_rgb2\_568\_106\_rev 5', mRNA sequence  
J gi|89860456|gb|DY716579.1|DY716579 EST\_ssal\_rgb2\_72318 ssalrgb2 mixed\_tissue Salmo salar cDNA Salmo salar cDNA clone ssal\_rgb2\_617\_064\_fwd 3', mRNA sequence  
K gi|117506109|gb|EG837868.1|EG837868 EST\_ssal\_eve\_12373 ssaleve thyroid Salmo salar cDNA Salmo salar cDNA clone ssal\_eve\_516\_319\_fwd 3', mRNA sequence  
L gi|117488162|gb|EG820379.1|EG820379 EST\_ssal\_evd\_22321 ssalevd thymus Salmo salar cDNA Salmo salar cDNA clone ssal\_evd\_528\_337\_fwd 3', mRNA sequence  
M gi|85016192|gb|DW544848.1|DW544848 EST\_ssal\_rgb2\_9267 rgb2 Salmo salar cDNA clone ssal\_rgb2\_515\_367\_rev 5', mRNA sequence  
N gi|117824196|gb|EG896892.1|EG896892 EST\_ssal\_evf\_5704 ssalevf mixed\_tissue Salmo salar cDNA Salmo salar cDNA clone ssal\_evf\_505\_366\_fwd 3', mRNA sequence  
O gi|85036578|gb|DW564835.1|DW564835 EST\_ssal\_rgb2\_29254 rgb2 Salmo salar cDNA clone ssal\_rgb2\_547\_184\_rev 5', mRNA sequence  
P gi|89855358|gb|DY711481.1|DY711481 EST\_ssal\_rgb2\_67220 ssalrgb2 mixed\_tissue Salmo salar cDNA Salmo salar cDNA clone ssal\_rgb2\_609\_099\_rev 5', mRNA sequence  
Q gi|117864950|gb|EG937646.1|EG937646 EST\_ssal\_evf\_21450 ssalevf mixed\_tissue Salmo salar cDNA Salmo salar cDNA clone ssal\_evf\_527\_083\_fwd 3', mRNA sequence  
R gi|117832402|gb|EG905098.1|EG905098 EST\_ssal\_evf\_53559 ssalevf mixed\_tissue Salmo salar cDNA Salmo salar cDNA clone ssal\_evf\_571\_292\_fwd 3', mRNA sequence  
S gi|81486210|gb|AM042103.1|AM042103 AM042103 Salmo salar head kidney Salmo salar cDNA clone HK2E5, mRNA sequence  
T gi|117454801|gb|EG787020.1|EG787020 EST\_ssal\_evd\_7638 ssalevd thymus Salmo salar cDNA Salmo salar cDNA clone ssal\_evd\_508\_356\_fwd 3', mRNA sequence  
U gi|117454610|gb|EG786829.1|EG786829 EST\_ssal\_evd\_7466 ssalevd thymus Salmo salar cDNA Salmo salar cDNA clone ssal\_evd\_508\_267\_fwd 3', mRNA sequence  
V gi|117486364|gb|EG818581.1|EG818581 EST\_ssal\_evd\_20704 ssalevd thymus Salmo salar cDNA Salmo salar cDNA clone ssal\_evd\_526\_272\_fwd 3', mRNA sequence  
W gi|117470250|gb|EG802469.1|EG802469 EST\_ssal\_evd\_59005 ssalevd thymus Salmo salar cDNA Salmo salar cDNA clone ssal\_evd\_579\_249\_fwd 3', mRNA sequence  
X gi|117833387|gb|EG906083.1|EG906083 EST\_ssal\_evf\_8632 ssalevf mixed\_tissue Salmo salar cDNA Salmo salar cDNA clone ssal\_evf\_509\_340\_fwd 3', mRNA sequence  
Y gi|117868221|gb|EG940917.1|EG940917 EST\_ssal\_evf\_17483 ssalevf mixed\_tissue Salmo salar cDNA Salmo salar cDNA clone ssal\_evf\_521\_346\_fwd 3', mRNA sequence  
Z gi|117841021|gb|EG913717.1|EG913717 EST\_ssal\_evf\_15504 ssalevf mixed\_tissue Salmo salar cDNA Salmo salar cDNA clone ssal\_evf\_519\_097\_fwd 3', mRNA sequence  
a gi|89856906|gb|DY713029.1|DY713029 EST\_ssal\_rgb2\_68768 ssalrgb2 mixed\_tissue Salmo salar cDNA Salmo salar cDNA clone ssal\_rgb2\_611\_241\_rev 5', mRNA sequence  
b gi|117488161|gb|EG820378.1|EG820378 EST\_ssal\_evd\_22320 ssalevd thymus Salmo salar cDNA Salmo salar cDNA clone ssal\_evd\_528\_337\_rev 5', mRNA sequence  
c gi|89866607|gb|DY722730.1|DY722730 EST\_ssal\_rgb2\_78469 ssalrgb2 mixed\_tissue Salmo salar cDNA Salmo salar cDNA clone ssal\_rgb2\_629\_051\_rev 5', mRNA sequence  
d gi|81487125|gb|AM042368.1|AM042368 AM042368 Salmo salar head kidney Salmo salar cDNA clone HK5H10, mRNA sequence  
e gi|75981039|gb|AM041670.1|AM041670 AM041670 pGemT-easy Atlantic salmon gill subtracted library (Aeromonas salmonicda infected salmon subtracted with uninfected) Salmo salar cDNA clone GT4B5, mRNA sequence  
f gi|85163469|gb|DW592251.1|DW592251 smus1-015AH01.z1\_#0573.2 DIAS\_SMUS Salmo salar cDNA 5', mRNA sequence  
g gi|75981056|gb|AM041687.1|AM041687 AM041687 pGemT-easy Atlantic salmon gill subtracted library (Aeromonas salmonicda infected salmon subtracted with uninfected) Salmo salar cDNA clone GT4D1, mRNA sequence  
h gi|85156515|gb|DW589158.1|DW589158 sbra1-008AD12.z1\_#0552.2 DIAS\_SBRA Salmo salar cDNA 5', mRNA sequence  
i gi|75981098|gb|AM041729.1|AM041729 AM041729 pGemT-easy Atlantic salmon gill subtracted library (Aeromonas salmonicda infected salmon subtracted with uninfected) Salmo salar cDNA clone GT4G5, mRNA sequence  
j gi|24382523|gb|CA052280.1|CA052280 ssalrga514363 mixed\_tissue Salmo salar cDNA, mRNA sequence  
k gi|89830497|gb|DY692789.1|DY692789 EST\_ssal\_plnb\_5699 ssalplnb mixed\_tissue Salmo salar cDNA Salmo salar cDNA clone ssal\_plnb\_027\_140\_fwd 3', mRNA sequence  
l gi|89884087|gb|DY740210.1|DY740210 EST\_ssal\_rgb2\_95949 ssalrgb2 mixed\_tissue Salmo salar cDNA Salmo salar cDNA clone ssal\_rgb2\_657\_038\_rev 5', mRNA sequence  
m gi|117847387|gb|EG920083.1|EG920083 EST\_ssal\_evf\_58972 ssalevf mixed\_tissue Salmo salar cDNA Salmo salar cDNA clone ssal\_evf\_579\_095\_fwd 3', mRNA sequence  
n gi|117470251|gb|EG802470.1|EG802470 EST\_ssal\_evd\_59006 ssalevd thymus Salmo salar cDNA Salmo salar cDNA clone ssal\_evd\_579\_249\_rev 5', mRNA sequence  
o gi|24385327|gb|CA055084.1|CA055084 ssalrgb503157 mixed\_tissue Salmo salar cDNA, mRNA sequence  
p gi|85018714|gb|DW547370.1|DW547370 EST\_ssal\_rgb2\_11789 rgb2 Salmo salar cDNA clone ssal\_rgb2\_519\_355\_fwd 3', mRNA sequence  
q gi|117506110|gb|EG837869.1|EG837869 EST\_ssal\_eve\_12374 ssaleve thyroid Salmo salar cDNA Salmo salar cDNA clone ssal\_eve\_516\_319\_rev 5', mRNA sequence  
r gi|85049532|gb|DW577710.1|DW577710 EST\_ssal\_rgb2\_42129 rgb2 Salmo salar cDNA clone ssal\_rgb2\_568\_106\_fwd 3', mRNA sequence  
s gi|89876895|gb|DY733018.1|DY733018 EST\_ssal\_rgb2\_88757 ssalrgb2 mixed\_tissue Salmo salar cDNA Salmo salar cDNA clone ssal\_rgb2\_645\_068\_rev 5', mRNA sequence  
t gi|117824197|gb|EG896893.1|EG896893 EST\_ssal\_evf\_5705 ssalevf mixed\_tissue Salmo salar cDNA Salmo salar cDNA clone ssal\_evf\_505\_366\_rev 5', mRNA sequence  
u gi|29323068|gb|CB511842.1|CB511842 ssalrgb547374 mixed\_tissue Salmo salar cDNA, mRNA sequence  
v gi|117847388|gb|EG920084.1|EG920084 EST\_ssal\_evf\_58973 ssalevf mixed\_tissue Salmo salar cDNA Salmo salar cDNA clone ssal\_evf\_579\_095\_rev 5', mRNA sequence  
w gi|29322829|gb|CB511603.1|CB511603 ssalrgb546037 mixed\_tissue Salmo salar cDNA, mRNA sequence  
x gi|89830498|gb|DY692790.1|DY692790 EST\_ssal\_plnb\_5700 ssalplnb mixed\_tissue Salmo salar cDNA Salmo salar cDNA clone ssal\_plnb\_027\_140\_rev 5', mRNA sequence  
y gi|117566938|gb|EG887914.1|EG887914 EST\_ssal\_evf\_46024 ssalevf mixed\_tissue Salmo salar cDNA Salmo salar cDNA clone ssal\_evf\_561\_196\_fwd 3', mRNA sequence  
z gi|117566939|gb|EG887915.1|EG887915 EST\_ssal\_evf\_46025 ssalevf mixed\_tissue Salmo salar cDNA Salmo salar cDNA clone ssal\_evf\_561\_196\_rev 5', mRNA sequence  
A gi|117454033|gb|EG786252.1|EG786252 EST\_ssal\_evd\_6946 ssalevd thymus Salmo salar cDNA Salmo salar cDNA clone ssal\_evd\_507\_381\_rev 5', mRNA sequence  
B gi|117821058|gb|EG893754.1|EG893754 EST\_ssal\_evf\_51280 ssalevf mixed\_tissue Salmo salar cDNA Salmo salar cDNA clone ssal\_evf\_568\_244\_rev 5', mRNA sequence  
C gi|117456706|gb|EG788925.1|EG788925 EST\_ssal\_evd\_9352 ssalevd thymus Salmo salar cDNA Salmo salar cDNA clone ssal\_evd\_511\_100\_rev 5', mRNA sequence  
D gi|117454800|gb|EG787019.1|EG787019 EST\_ssal\_evd\_7637 ssalevd thymus Salmo salar cDNA Salmo salar cDNA clone ssal\_evd\_508\_356\_rev 5', mRNA sequence  
E gi|84984864|gb|DW535214.1|DW535214 EST\_ssal\_plnb\_3850 plnb Salmo salar cDNA clone ssal\_plnb\_021\_324\_fwd 3', mRNA sequence  
F gi|117854877|gb|EG927573.1|EG927573 EST\_ssal\_evf\_29973 ssalevf mixed\_tissue Salmo salar cDNA Salmo salar cDNA clone ssal\_evf\_539\_298\_rev 5', mRNA sequence  
G gi|117834965|gb|EG907661.1|EG907661 EST\_ssal\_evf\_10052 ssalevf mixed\_tissue Salmo salar cDNA Salmo salar cDNA clone ssal\_evf\_511\_308\_rev 5', mRNA sequence  
H gi|24464124|gb|BQ036743.1|BQ036743 SS2-0452 Atlantic Salmon spleen SSH Salmo salar cDNA clone SS2-0452 similar to other reported ESTs, mRNA sequence  
I gi|24392897|gb|CA062654.1|CA062654 ssalrgb524105 mixed\_tissue Salmo salar cDNA, mRNA sequence  
J gi|24353967|gb|CA047797.1|CA047797 ssalga004050 head Salmo salar cDNA, mRNA sequence  
K gi|45649293|gb|CK990324.1|CK990324 ssalga502025 head Salmo salar cDNA, mRNA sequence  
L gi|29311975|gb|CB500749.1|CB500749 ssalga505095 head Salmo salar cDNA, mRNA sequence  
M gi|117481374|gb|EG813591.1|EG813591 EST\_ssal\_evd\_33552 ssalevd thymus Salmo salar cDNA Salmo salar cDNA clone ssal\_evd\_544\_260\_rev 5', mRNA sequence  
N gi|117835449|gb|EG908145.1|EG908145 EST\_ssal\_evf\_10488 ssalevf mixed\_tissue Salmo salar cDNA Salmo salar cDNA clone ssal\_evf\_512\_158\_fwd 3', mRNA sequence  
O gi|117835450|gb|EG908146.1|EG908146 EST\_ssal\_evf\_10489 ssalevf mixed\_tissue Salmo salar cDNA Salmo salar cDNA clone ssal\_evf\_512\_158\_rev 5', mRNA sequence  
P gi|117485641|gb|EG817858.1|EG817858 EST\_ssal\_evd\_20052 ssalevd thymus Salmo salar cDNA Salmo salar cDNA clone ssal\_evd\_525\_312\_fwd 3', mRNA sequence  
Q gi|85045476|gb|DW573654.1|DW573654 EST\_ssal\_rgb2\_38073 rgb2 Salmo salar cDNA clone ssal\_rgb2\_561\_362\_rev 5', mRNA sequence  
R gi|85045477|gb|DW573655.1|DW573655 EST\_ssal\_rgb2\_38074 rgb2 Salmo salar cDNA clone ssal\_rgb2\_561\_362\_fwd 3', mRNA sequence  
S gi|117864949|gb|EG937645.1|EG937645 EST\_ssal\_evf\_21449 ssalevf mixed\_tissue Salmo salar cDNA Salmo salar cDNA clone ssal\_evf\_527\_083\_rev 5', mRNA sequence  
T gi|57125295|gb|CX356736.1|CX356736 ssalrgb524105\_rev\_0 mixed\_tissue Salmo salar cDNA, mRNA sequence  
U gi|117449246|gb|EG781465.1|EG781465 EST\_ssal\_evd\_38103 ssalevd thymus Salmo salar cDNA Salmo salar cDNA clone ssal\_evd\_550\_340\_rev 5', mRNA sequence  
V gi|89876239|gb|DY732362.1|DY732362 EST\_ssal\_rgb2\_88101 ssalrgb2 mixed\_tissue Salmo salar cDNA Salmo salar cDNA clone ssal\_rgb2\_644\_023\_fwd 3', mRNA sequence  
W gi|89876238|gb|DY732361.1|DY732361 EST\_ssal\_rgb2\_88100 ssalrgb2 mixed\_tissue Salmo salar cDNA Salmo salar cDNA clone ssal\_rgb2\_644\_023\_rev 5', mRNA sequence  
X gi|117845171|gb|EG917867.1|EG917867 EST\_ssal\_evf\_56977 ssalevf mixed\_tissue Salmo salar cDNA Salmo salar cDNA clone ssal\_evf\_576\_158\_rev 5', mRNA sequence  
Y gi|85029983|gb|DW558639.1|DW558639 EST\_ssal\_rgb2\_23058 rgb2 Salmo salar cDNA clone ssal\_rgb2\_537\_175\_fwd 3', mRNA sequence  
Z gi|89856907|gb|DY713030.1|DY713030 EST\_ssal\_rgb2\_68769 ssalrgb2 mixed\_tissue Salmo salar cDNA Salmo salar cDNA clone ssal\_rgb2\_611\_241\_fwd 3', mRNA sequence  
a gi|117845518|gb|EG918214.1|EG918214 EST\_ssal\_evf\_57290 ssalevf mixed\_tissue Salmo salar cDNA Salmo salar cDNA clone ssal\_evf\_576\_322\_rev 5', mRNA sequence  
b gi|117472618|gb|EG804837.1|EG804837 EST\_ssal\_evd\_5673 ssalevd thymus Salmo salar cDNA Salmo salar cDNA clone ssal\_evd\_506\_102\_rev 5', mRNA sequence  
c gi|25997315|gb|CA768060.1|CA768060 ssalplnb504079 gut Salmo salar cDNA, mRNA sequence  
d gi|24390339|gb|CA060096.1|CA060096 ssalrgb513007 mixed\_tissue Salmo salar cDNA, mRNA sequence  
e gi|85036579|gb|DW564836.1|DW564836 EST\_ssal\_rgb2\_29255 rgb2 Salmo salar cDNA clone ssal\_rgb2\_547\_184\_fwd 3', mRNA sequence  
f gi|117862488|gb|EG935184.1|EG935184 EST\_ssal\_evf\_19233 ssalevf mixed\_tissue Salmo salar cDNA Salmo salar cDNA clone ssal\_evf\_524\_093\_rev 5', mRNA sequence  
g gi|117525621|gb|EG857348.1|EG857348 EST\_ssal\_eve\_50904 ssaleve thyroid Salmo salar cDNA Salmo salar cDNA clone ssal\_eve\_569\_125\_rev 5', mRNA sequence  
h gi|117833386|gb|EG906082.1|EG906082 EST\_ssal\_evf\_8631 ssalevf mixed\_tissue Salmo salar cDNA Salmo salar cDNA clone ssal\_evf\_509\_340\_rev 5', mRNA sequence  
i gi|117838807|gb|EG911503.1|EG911503 EST\_ssal\_evf\_13511 ssalevf mixed\_tissue Salmo salar cDNA Salmo salar cDNA clone ssal\_evf\_516\_223\_rev 5', mRNA sequence  
j gi|24352014|gb|CA045844.1|CA045844 ssalga003033 head Salmo salar cDNA, mRNA sequence  
k gi|29312810|gb|CB501584.1|CB501584 ssalga508004 head Salmo salar cDNA, mRNA sequence  
l gi|29320703|gb|CB509477.1|CB509477 ssalnwh503132 whole Salmo salar cDNA, mRNA sequence  
m gi|29322645|gb|CB511419.1|CB511419 ssalnwh502365 whole Salmo salar cDNA, mRNA sequence  
n gi|29312852|gb|CB501626.1|CB501626 ssalga508098 head Salmo salar cDNA, mRNA sequence  
o gi|117530468|gb|EG861924.1|EG861924 EST\_ssal\_eve\_55022 ssaleve thyroid Salmo salar cDNA Salmo salar cDNA clone ssal\_eve\_575\_001\_rev 5', mRNA sequence  
p gi|24337520|gb|CA037955.1|CA037955 ssallna001030 liver Salmo salar cDNA, mRNA sequence  
q gi|117299631|gb|EG649443.1|EG649443 SGP318427 Atlantic salmon normalized Muscle cDNA library Salmo salar cDNA clone MUSCLE4-D7 5', mRNA sequence  
r gi|117865789|gb|EG938485.1|EG938485 EST\_ssal\_evf\_22205 ssalevf mixed\_tissue Salmo salar cDNA Salmo salar cDNA clone ssal\_evf\_529\_087\_rev 5', mRNA sequence  
s gi|117865749|gb|EG938445.1|EG938445 EST\_ssal\_evf\_22169 ssalevf mixed\_tissue Salmo salar cDNA Salmo salar cDNA clone ssal\_evf\_529\_068\_fwd 3', mRNA sequence  
t gi|117869762|gb|EG942458.1|EG942458 EST\_ssal\_evf\_25781 ssalevf mixed\_tissue Salmo salar cDNA Salmo salar cDNA clone ssal\_evf\_534\_053\_rev 5', mRNA sequence  
u gi|117865748|gb|EG938444.1|EG938444 EST\_ssal\_evf\_22168 ssalevf mixed\_tissue Salmo salar cDNA Salmo salar cDNA clone ssal\_evf\_529\_068\_rev 5', mRNA sequence  
v gi|117858706|gb|EG931402.1|EG931402 EST\_ssal\_evf\_33419 ssalevf mixed\_tissue Salmo salar cDNA Salmo salar cDNA clone ssal\_evf\_544\_168\_rev 5', mRNA sequence  
w gi|117532330|gb|EG863775.1|EG863775 EST\_ssal\_eve\_48887 ssaleve thyroid Salmo salar cDNA Salmo salar cDNA clone ssal\_eve\_566\_216\_rev 5', mRNA sequence  
x gi|25996910|gb|CA767655.1|CA767655 ssalmgf004021 gut Salmo salar cDNA, mRNA sequence  
y gi|117298922|gb|EG648734.1|EG648734 SGP259235 Atlantic salmon normalized Muscle cDNA library Salmo salar cDNA clone NM1-0960 5', mRNA sequence  
z gi|29311687|gb|CB500461.1|CB500461 ssalga506031 head Salmo salar cDNA, mRNA sequence  
A gi|89850201|gb|DY706324.1|DY706324 EST\_ssal\_rgb2\_62063 ssalrgb2 mixed\_tissue Salmo salar cDNA Salmo salar cDNA clone ssal\_rgb2\_599\_320\_fwd 3', mRNA sequence  
B gi|117490245|gb|EG822462.1|EG822462 EST\_ssal\_evd\_24197 ssalevd thymus Salmo salar cDNA Salmo salar cDNA clone ssal\_evd\_531\_168\_rev 5', mRNA sequence  
C gi|117548983|gb|EG880428.1|EG880428 EST\_ssal\_eve\_23746 ssaleve thyroid Salmo salar cDNA Salmo salar cDNA clone ssal\_eve\_532\_138\_rev 5', mRNA sequence  
D gi|117852851|gb|EG925547.1|EG925547 EST\_ssal\_evf\_28149 ssalevf mixed\_tissue Salmo salar cDNA Salmo salar cDNA clone ssal\_evf\_537\_123\_rev 5', mRNA sequence  
E gi|117841020|gb|EG913716.1|EG913716 EST\_ssal\_evf\_15503 ssalevf mixed\_tissue Salmo salar cDNA Salmo salar cDNA clone ssal\_evf\_519\_097\_rev 5', mRNA sequence  
F gi|117452537|gb|EG784756.1|EG784756 EST\_ssal\_evd\_41065 ssalevd thymus Salmo salar cDNA Salmo salar cDNA clone ssal\_evd\_555\_001\_fwd 3', mRNA sequence  
G gi|117452538|gb|EG784757.1|EG784757 EST\_ssal\_evd\_41066 ssalevd thymus Salmo salar cDNA Salmo salar cDNA clone ssal\_evd\_555\_001\_rev 5', mRNA sequence  
H gi|117845340|gb|EG918036.1|EG918036 EST\_ssal\_evf\_57130 ssalevf mixed\_tissue Salmo salar cDNA Salmo salar cDNA clone ssal\_evf\_576\_241\_rev 5', mRNA sequence  
I gi|117845341|gb|EG918037.1|EG918037 EST\_ssal\_evf\_57131 ssalevf mixed\_tissue Salmo salar cDNA Salmo salar cDNA clone ssal\_evf\_576\_241\_fwd 3', mRNA sequence  
J gi|117486365|gb|EG818582.1|EG818582 EST\_ssal\_evd\_20705 ssalevd thymus Salmo salar cDNA Salmo salar cDNA clone ssal\_evd\_526\_272\_rev 5', mRNA sequence  
K gi|117446641|gb|EG778860.1|EG778860 EST\_ssal\_evd\_5358 ssalevd thymus Salmo salar cDNA Salmo salar cDNA clone ssal\_evd\_505\_318\_rev 5', mRNA sequence  
L gi|117458008|gb|EG790227.1|EG790227 EST\_ssal\_evd\_10524 ssalevd thymus Salmo salar cDNA Salmo salar cDNA clone ssal\_evd\_512\_334\_rev 5', mRNA sequence  
M gi|24394140|gb|CA063897.1|CA063897 ssalrga511289 mixed\_tissue Salmo salar cDNA, mRNA sequence  
N gi|117825368|gb|EG898064.1|EG898064 EST\_ssal\_evf\_37158 ssalevf mixed\_tissue Salmo salar cDNA Salmo salar cDNA clone ssal\_evf\_549\_197\_rev 5', mRNA sequence  
O gi|117832391|gb|EG905087.1|EG905087 EST\_ssal\_evf\_53558 ssalevf mixed\_tissue Salmo salar cDNA Salmo salar cDNA clone ssal\_evf\_571\_292\_rev 5', mRNA sequence  
P gi|117856687|gb|EG929383.1|EG929383 EST\_ssal\_evf\_31603 ssalevf mixed\_tissue Salmo salar cDNA Salmo salar cDNA clone ssal\_evf\_541\_372\_rev 5', mRNA sequence  
Q gi|117858555|gb|EG931251.1|EG931251 EST\_ssal\_evf\_33284 ssalevf mixed\_tissue Salmo salar cDNA Salmo salar cDNA clone ssal\_evf\_544\_100\_rev 5', mRNA sequence  
R gi|117868232|gb|EG940928.1|EG940928 EST\_ssal\_evf\_17484 ssalevf mixed\_tissue Salmo salar cDNA Salmo salar cDNA clone ssal\_evf\_521\_346\_rev 5', mRNA sequence  
S gi|117484404|gb|EG816621.1|EG816621 EST\_ssal\_evd\_18938 ssalevd thymus Salmo salar cDNA Salmo salar cDNA clone ssal\_evd\_524\_119\_rev 5', mRNA sequence  
T gi|117484405|gb|EG816622.1|EG816622 EST\_ssal\_evd\_18939 ssalevd thymus Salmo salar cDNA Salmo salar cDNA clone ssal\_evd\_524\_119\_fwd 3', mRNA sequence  
U gi|117444497|gb|EG776720.1|EG776720 EST\_ssal\_evd\_51833 ssalevd thymus Salmo salar cDNA Salmo salar cDNA clone ssal\_evd\_569\_325\_rev 5', mRNA sequence  
V gi|117444498|gb|EG776721.1|EG776721 EST\_ssal\_evd\_51834 ssalevd thymus Salmo salar cDNA Salmo salar cDNA clone ssal\_evd\_569\_325\_fwd 3', mRNA sequence  
W gi|117852918|gb|EG925614.1|EG925614 EST\_ssal\_evf\_28210 ssalevf mixed\_tissue Salmo salar cDNA Salmo salar cDNA clone ssal\_evf\_537\_154\_rev 5', mRNA sequence  
X gi|89850868|gb|DY706991.1|DY706991 EST\_ssal\_rgb2\_62730 ssalrgb2 mixed\_tissue Salmo salar cDNA Salmo salar cDNA clone ssal\_rgb2\_601\_122\_fwd 3', mRNA sequence  
Y gi|117443459|gb|EG775682.1|EG775682 EST\_ssal\_evd\_50898 ssalevd thymus Salmo salar cDNA Salmo salar cDNA clone ssal\_evd\_568\_215\_rev 5', mRNA sequence  
Z gi|89872320|gb|DY728443.1|DY728443 EST\_ssal\_rgb2\_84182 ssalrgb2 mixed\_tissue Salmo salar cDNA Salmo salar cDNA clone ssal\_rgb2\_637\_368\_fwd 3', mRNA sequence  
a gi|89872319|gb|DY728442.1|DY728442 EST\_ssal\_rgb2\_84181 ssalrgb2 mixed\_tissue Salmo salar cDNA Salmo salar cDNA clone ssal\_rgb2\_637\_368\_rev 5', mRNA sequence  
b gi|117858087|gb|EG930783.1|EG930783 EST\_ssal\_evf\_32862 ssalevf mixed\_tissue Salmo salar cDNA Salmo salar cDNA clone ssal\_evf\_543\_264\_rev 5', mRNA sequence  
c gi|85055964|gb|DW584142.1|DW584142 EST\_ssal\_rgb2\_48561 rgb2 Salmo salar cDNA clone ssal\_rgb2\_578\_165\_fwd 3', mRNA sequence

4 SNPs detected

A B C D E F G H I J K L M N O P Q R S T U V W X Y Z a b c d e f g h i j k l m n o p q r s t u v w x y z A B C D E F G H I J K L M N O P Q R S T U V W X Y Z a b c d e f g h i j k l m n o p q r s t u v w x y z A B C D E F G H I J K L M N O P Q R S T U V W X Y Z a b c d e f g h i j k l m n o p q r s t u v w x y z A B C D E F G H I J K L M N O P Q R S T U V W X Y Z a b c  cosegregation weighted

872 . . . . . . . . . . . . . . . . . . . . . . . . T C C T T . . C C . . C C C C C C C T T T T T T T C C C C C C C C C T C C C T T C C C C C C C T T T C C C C C T C C C . C C C C C C T C C C T C C C C T C C T T T T T T T T T . C C C C C C C C C C C C C C C C C C C C C C C C C C C T T C C T T T T T T T T T . C C C C C T T C C C T T T T C C C C C C C C C T T C C C C C T C   1/4 20.81
983 . . . . . . . . . . . . . . . . . . . . . . . . . . . . . . . . . . . . . . . . . . . . . . . . . . . . . . . . . . . T T T . T . . T - - - - T T T - - - - - T - - T T T T T T - T T - T T T T - - - T T - T T T T T T T T T . - - - - - - - - - - - - - - - - - - - - - - - - - - - T T T T T T T T T T T T T . T T T T - T T - - - T T T T - - - - - - - T T T T - T T T T T T   2/4 32.70
984 . . . . . . . . . . . . . . . . . . . . . . . . . . . . . . . . . . . . . . . . . . . . . . . . . . . . . . . . . . . T T T . T . . G - - - - T T T - - - - - T - - T T T T T T - T T - T T T T - - - T T - T T T T T T T T T . - - - - - - - - - - - - - - - - - - - - - - - - - - - T T T T T T T T T T T T T . T T T T - T T - - - T T T T - - - - - - - T T T T - T T T T T T   1/4 16.35
1047 . . . . . . . . . . . . . . . . . . . . . . . . . . . . . . . . . . . . . . . . . . . . . . . . . . . . . . . . . . . . . . . . . . . . . . . T . T . . . G G . G G T T . T T T G T T G T T T T G G G T T G T T T T T T T T T . G G G G G G G G G G G G G G G G G G G G G G G G G G G T T T T T T T T T T T T T T T T T T G T T G G G T T T T G G G G G G G T T T T G T T T T T T   2/4 28.92
